# Tenfibgen Ligand Nanoencapsulation Delivers Bi-Functional Anti-CK2 RNAi Oligomer to Key Sites for Prostate Cancer Targeting Using Human Xenograft Tumors in Mice

**DOI:** 10.1371/journal.pone.0109970

**Published:** 2014-10-15

**Authors:** Janeen H. Trembley, Gretchen M. Unger, Vicci L. Korman, Md. Joynal Abedin, Lucas P. Nacusi, Rachel I. Vogel, Joel W. Slaton, Betsy T. Kren, Khalil Ahmed

**Affiliations:** 1 Research Service, Minneapolis VA Health Care System, Minneapolis, Minnesota, United States of America; 2 Department of Laboratory Medicine and Pathology, University of Minnesota, Minneapolis, Minnesota, United States of America; 3 Masonic Cancer Center, University of Minnesota, Minneapolis, Minnesota, United States of America; 4 GeneSegues Inc., Chaska, Minnesota, United States of America; 5 Department of Medicine, University of Minnesota, Minneapolis, Minnesota, United States of America; 6 Department of Urology, University of Minnesota, Minneapolis, Minnesota, United States of America; University of Illinois, United States of America

## Abstract

Protected and specific delivery of nucleic acids to malignant cells remains a highly desirable approach for cancer therapy. Here we present data on the physical and chemical characteristics, mechanism of action, and pilot therapeutic efficacy of a tenfibgen (TBG)-shell nanocapsule technology for tumor-directed delivery of single stranded DNA/RNA chimeric oligomers targeting CK2αα' to xenograft tumors in mice. The sub-50 nm size TBG nanocapsule (s50-TBG) is a slightly negatively charged, uniform particle of 15 - 20 nm size which confers protection to the nucleic acid cargo. The DNA/RNA chimeric oligomer (RNAi-CK2) functions to decrease CK2αα' expression levels via both siRNA and antisense mechanisms. Systemic delivery of s50-TBG-RNAi-CK2 specifically targets malignant cells, including tumor cells in bone, and at low doses reduces size and CK2-related signals in orthotopic primary and metastatic xenograft prostate cancer tumors. In conclusion, the s50-TBG nanoencapsulation technology together with the chimeric oligomer targeting CK2αα' offer significant promise for systemic treatment of prostate malignancy.

## Introduction

Across the spectrum of malignant disease, effective therapy for advanced and/or metastatic cancer remains a critical goal in efforts to reduce cancer-related mortality. Nucleic acid-based cancer therapy continues to hold significant promise for gene-specific, effective, and low-toxicity disease treatment which has the additional potential of overcoming therapeutic resistance frequently observed in aggressive disease. Both antisense and siRNA-based therapeutics have entered into clinical trials with very moderate success [1]–[4]. Key considerations for the systemic use of short linear nucleic acids as a therapeutic moiety include that they are directed specifically to tumor cells in a protected manner, effectively cross cell membranes and engage the cell machinery. Numerous approaches incorporating these concepts have demonstrated utility in mouse models including, e.g., PSMA-targeted aptamer-siRNA chimeras, Her2-targeted single chain fragmented antibody-protamine fusion protein mediated siRNA delivery, and transferrin receptor-targeted cyclodextrin-based polymer mediated siRNA delivery [1], [5], [6]. Our approach to effectively deliver nucleic acid therapeutics to tumors utilizes a sub-50 nm size nanocapsule comprising the ligand tenfibgen (TBG), the carboxy-terminal fibrinogen globe domain of tenascin-C. TBG forms the protein ligand shell of the nanocapsule and allows receptor-mediated targeting to cancer cells via tenascin receptors, which are elevated in these cells [7]–[12]. TBG nanocapsules are specifically taken up by tumor cells and tumor-derived microvessels but not by normal cells or vessels [13]–[15].

CK2 (formerly casein kinase II or CKII) is an ubiquitous protein Ser/Thr kinase with a heterotetrameric structure consisting of two catalytic subunits (42 kDa α and/or 38 kDa α′) and two regulatory subunits (28 kDa β) in α_2_β_2_, αα′β_2_, or α′_2_β_2_ configurations. CK2 phosphorylates a large number of substrates with various functions relating to cell growth and proliferation, is constitutively active, and is essential for survival [16]–[22]. Moreover, CK2 has been found to be upregulated in all cancers examined, where one of its functions is to suppress apoptosis [18], [23]–[29]. We have previously established the efficacy of our 20–mer nucleic acid sequence for targeting both CK2α and CK2α′. We have used this sequence as an antisense oligonucleotide delivered systemically to mice carrying orthotopic PCa tumors, as an siRNA cargo for s50-TBG nanocapsules delivered to cultured prostate cancer (PCa) cells, and as a single stranded RNAi-CK2 cargo for s50-TBG nanocapsules delivered systemically to mice carrying head and neck squamous cell carcinoma (HNSCC) xenografts [15], [30]–[32]. Here we expand on the mechanism and treatment utility of both the s50-TBG nanocapsule and the RNAi-CK2 oligomer using human prostate cancer xenograft models initiated in mice in therapeutically relevant sites. We present data characterizing the s50-TBG nanocapsule and its systemic delivery as proof-of-concept for ability to target important *in vivo* PCa tumor growth sites as well as the efficacy of using RNAi-mediated downregulation of CK2 as a therapeutic approach.

## Results

### Tenfibgen nanocapsule design and stability

**Figure 1:**
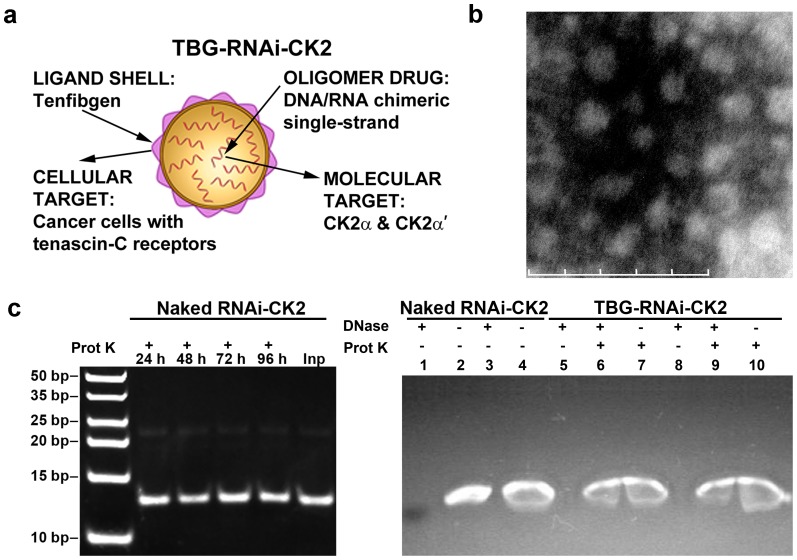
Nanocapsule design, morphology, cargo stability, and cargo protection. (**a**) Cartoon depiction of nanocapsule design. (**b**) Transmission electron micrograph of s50-TBG-RNAi-CK2 nanocapsules for *in vivo* studies. Magnification 230,000×. Scale bar 100 nm. (**c**) Left panel: Naked RNAi-CK2 oligomer was digested with proteinase K for 24 to 96 h. Inp, undigested input oligomer. Right panel: Naked and s50-TBG encapsulated RNAi-CK2 oligomers were digested with DNase followed by proteinase K as indicated above the panel. Lanes 1 & 2, naked RNAi-CK2; 3 & 4, naked RNAi-CK2 with TBG-sugar nanocapsules included in the digestion; 5 - 7, *in vitro* use formulation of s50-TBG-RNAi-CK2; 8 – 10, *in vivo* use formulation of s50-TBG-RNAi-CK2.

To meet our goal of decreasing CK2 expression specifically in malignant prostate cells, a single-stranded DNA/RNA chimeric molecule (RNAi-CK2) targeting the CK2αα' subunit transcripts was condensed and encapsulated within a protein shell composed of TBG. TBG nanocapsules are typically 15–20 nm in size, and enter tumor cells via tenascin receptors using the lipid raft-mediated caveolar pathway [13], [15]. Characteristics and morphology of TBG-RNAi-CK2 nanocapsules as well as the structure and sequence of RNAi-CK2 are summarized in Table 1 and **Figure 1a,b**. Stability analysis of naked RNAi-CK2 chimeric oligomer and s50-TBG-RNAi-CK2 nanocapsules (**Fig. 1c**) demonstrates that after proteinase K digestion of naked RNAi-CK2 oligomer (left panel) or s50-TBG-RNAi-CK2 nanocapsules (right panel), the oligomer cargo remains intact. Moreover, treatment of nanocapsules with DNase prior to proteinase K digestion shows that the nucleic acid cargo is fully protected by the encapsulation procedure (**Fig. 1c**, right panel).

**Table 1:** In Vivo Nanocapsule Characteristics and Information.

Shell Ligand	Particle Size (nm)^1^	Zeta Potential (meV)^2^	Morphology^3^	Cargo^4^	Sequence of RNAi-CK2^5^
Tenfibgen (TBG) 27 kDa	17.6±2.5	−6.2±3.1	Uniform, Single Capsules	RNAi-CK2	5′ATACAACCCAAACTccacau(propyl)3′ (antisense/guide strand)

1 Mean ± SD of the average elliptical diameter determined from TEM micrographs (magnification 230,000×) of 20 nanocapsules from two different grid preparations.

2 Average surface charge measured by DLS from two different preparations across a 20 volt potential in 1 mM KCl at 2 µg/ml. Data shown is the mean ± SE of 15 independent measurements.

3 Morphology of all nanocapsules determined by visual AFM and TEM observation as uniform, single capsules.

4 TBG-RNAi-CK2 encapsulation efficiency mean of 79.8±6.1% observed by Burton analysis relative to unencapsulated oligomer.

5 Upper case letters represent phosphodiester DNA bases; lower case letters represent 2′ *O*-methyl RNA bases.

### Potential mechanisms of action for the RNAi-CK2 DNA/RNA chimeric oligomer

**Figure 2:**
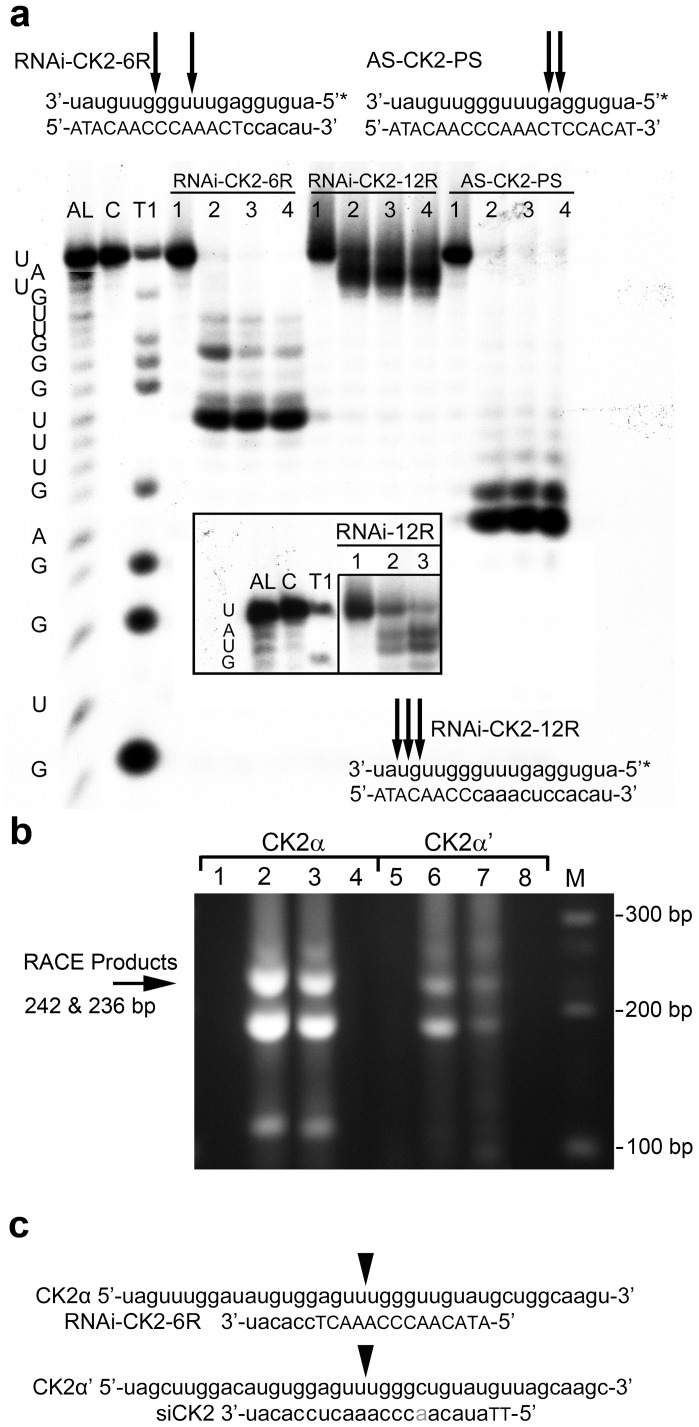
Characterization of potential mechanisms of CK2 expression downregulation by s50-TBG-RNAi-CK2. (**a**) RNase H1 substrate testing of different DNA/RNA composition forms of RNAi-CK2 oligomers. 5′ end-labeled (*) RNA probe was annealed with RNAi-CK2-6R, RNAi-CK2-12R or AS-CK2-PS complementary oligomers, then incubated for various periods of time with RNase H1. The letters on the left-hand side of the gel and inset represent the sequence ladders for the 5′ end-labeled RNA substrate. Sizing ladders are indicated as AL (alkaline lysis), C (minus RNase T1), and T1 (RNase T1 cleavage). RNase H1 digestion times are 0, 30, 60 and 120 s (lanes 1–4, respectively). The identity of the test oligomer is indicated above the lanes. The inset shows a lighter exposure of the RNAi-CK2-12R RNase H1 reaction products. Oligomers complemented with the labeled RNA probe are shown with arrowheads indicating major cleavage sites. For the RNAi-CK2-6R and RNAi-CK2-12R oligomer sequences, lower case denotes 2′ *O*-methyl RNA residues and capital letters are conventional phosphodiester linked DNA residues. The AS-CK2-PS is a phosphorothioate linked DNA oligomer. (**b**) Detection of Ago2/RISC cleavage products produced by RNAi-CK2 transfection into PC3-LN4 cells. 5′ RNA ligase-mediated RACE was performed for the CK2α and CK2α' transcripts as outlined in online methods. Lanes 1 & 5, siControl transfected cells; 2 & 6, siCK2 transfected cells; 3 & 7, RNAi-CK2 transfected cells; 4 & 8, no cDNA water controls; M, DNA size markers. The predicted RACE products (CK2α, 242 bp; CK2α', 236 bp) are indicated at left, and the gene specific primers used are indicated above the lanes. (**c**) Mapping of the RACE cleavage site was obtained by sequencing the products obtained in (b). The mRNA sequence is shown in lowercase, the transfected oligomer is depicted below the mRNA, and cleavage sites are indicated by an arrowhead.

The single stranded DNA/RNA oligomer cargo RNAi-CK2 could function to decrease CK2 expression through antisense- and/or siRNA-based mechanisms. To address this, we first investigated whether our RNAi-CK2 oligomer design consisting of 14 standard phosphodiester DNA residues at the 5′ end, 6 2′ *O*-methyl RNA residues at the 3′ end, and a terminal propyl linker (labeled RNAi-CK2-6R in **Fig. 2a**) could serve as a stimulus for RNase H1 activity. For comparison, we included the same sequence consisting entirely of phosphorothioate (PS) modified DNA residues (AS-CK2-PS) as well as an oligomer design with 8 standard DNA residues at the 5′ end, 12 2′ *O*-methyl RNA residues at the 3′ end, and a terminal propyl linker (RNAi-CK2-12R). The data in **Figure 2a** show that the RNAi-CK2 oligomer with 14 DNA residues (RNAi-CK2-6R) serves as an effective stimulus for RNase H1 activity. Throughout the remainder of the manuscript, RNAi-CK2-6R is referred to as RNAi-CK2 and is the therapeutic drug encapsulated in the TBG nanocapsules.

To examine the involvement of siRNA based mechanisms, RNA purified from TBG-RNAi-CK2 transfected PC3-LN4 [33] cells grown on tenascin-C/fibronectin (TnFn) was analyzed using a modified 5′ RNA ligase-mediated RACE technique to map any potential cleavage sites resulting from Ago2/RISC-based processing of the DNA/RNA chimeric oligomer. Purified RNA from cells transfected with siRNA to CK2 (same sequence target as RNAi-CK2) or a non-targeting control sequence were used as further controls. As shown in **Figure 2b**, cleavage products of CK2α and α′ transcripts were detected in both RNAi-CK2 and siCK2 transfected cells. Mapping of the cleavage sites by sequencing the RACE products confirmed the cleavage location in both transcripts at the predicted 10^th^ nucleotide from the 5′ end of the oligomer[34] (**Fig. 2c**). Together, these results indicate that RNAi-CK2 can down-regulate CK2α and CK2α' transcripts by RNase H and Ago2 mechanisms.

### Effects of TBG-RNAi-CK2 on malignant and benign cultured cells

**Figure 3:**
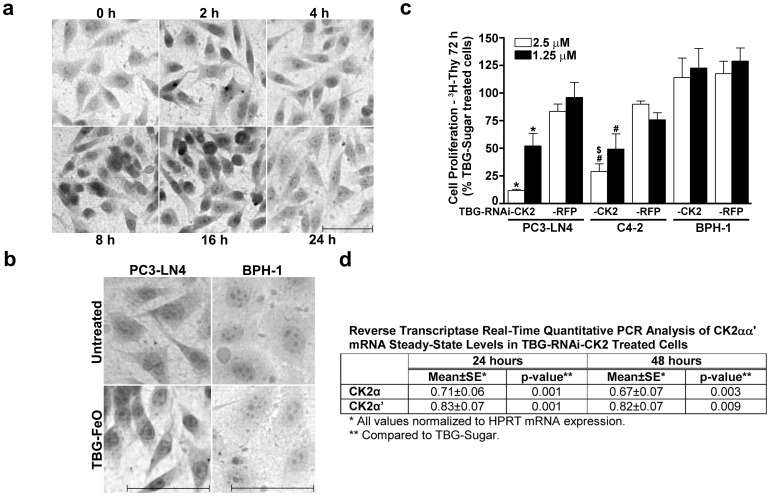
Cellular uptake of s50-TBG nanocapsules and effects of s50-TBG-RNAi-CK2 in benign and malignant prostate cells. (**a**) Uptake over 24 h of s50-TBG nanocapsules with FeO-dextran cargo in PC3-LN4 cells plated onto TnFn-3D. Cells were stained with DAB-enhanced Prussian blue for iron and counterstained with Fast Red. Scale bar 100 µm. (**b**) Malignant cell-specific uptake of s50-TBG nanocapsules. s50-TBG-FeO-dextran uptake was determined by iron staining at 8 h in PC3-LN4 and BPH-1 cells grown on TnFn- or laminin-coated nanofiber scaffolds, respectively. Scale bar 100 µm. (**c**) Cellular proliferation effects of s50-TBG-RNAi-CK2 treatment in benign and malignant prostate cells. PC3-LN4 and C4-2 grown on TnFn-3D and BPH-1 cells grown on laminin-3D in 96-well plates were treated with s50-TBG-RNAi-CK2 or control TBG nanocapsules containing RNAi-RFP-6R targeting Red Fluorescence Protein as indicated. ^3^H-thymidine was added after 48 h, and cells analyzed at 72 h post-nanocapsule addition. Results are expressed relative to treatment with s50-TBG-sugar nanocapsules. The s50-TBG nanocapsule cargo and cell lines used are indicated below the bars. Means ± SE are presented (n = 3 for all). *p<0.005 relative to s50-TBG-sugar and –RFP; # p<0.01 relative to TBG-sugar; $ p = 0.006 relative to TBG-RFP. (**d**) s50-TBG-RNAi-CK2 treatment reduced CK2α and CK2α' mRNA steady-state expression levels in PC3-LN4 cells. mRNA isolated from PC3-LN4 cells grown on TnFn 24 and 48 h after s50-TBG-RNAi-CK2 or –sugar treatment as indicated was analyzed by reverse transcriptase real-time quantitative PCR for CK2α and CK2α' expression. HPRT transcript was used to normalize expression levels. Means, SE and p-values are presented (24 h CK2α n = 5, CK2α' n = 6; 48 h CK2α n = 2, CK2α' n = 2).

We have consistently observed that s50-TBG nanocapsules deliver their cargo to the perinuclear region preceding nuclear entry, regardless of cancer cell type. For non-nucleic acid species, such as contrast agents, cargo is typically exported from nuclei to cytoplasm, where it accumulates in cytoplasmic organelles and is subsequently exported. To further characterize trafficking and cargo release of s50-TBG nanocapsules, malignant PC3-LN4 cells were cultured on TnFn coated 3-dimensional nanofiber scaffolds (TnFn-3D) to facilitate formation of the lipid rafts necessary for s50-TBG nanocapsule studies [15]. **Figure 3a** illustrates transport to the nucleus, cargo release, and export of FeO-dextran over a 24 h period of time after treatment of the cells with s50-TBG-FeO-dextran nanocapsules. In a similar study, a comparison of s50-TBG-FeO-dextran uptake 8 h after addition of nanocapsules to cells was performed in PC3-LN4 and benign prostatic hyperplasia (BPH-1) cells. The results demonstrate avid uptake of the TBG-FeO nanocapsules by malignant but not benign cells (**Fig. 3b**).

We examined the effects of s50-TBG-RNAi-CK2 on the proliferation of cultured prostate cells using the highly tumorigenic PC3-LN4 and C4-2 cell lines in comparison to BPH-1. Treatment of PC3-LN4 and C4-2 cells grown on TnFn-3D with s50-TBG-RNAi-CK2 for 3 days resulted in a significant loss of cell proliferation measured by [^3^H]-thymidine incorporation, whereas TBG-RNAi-CK2 treatment of BPH-1 cells grown on a laminin matrix did not reduce their proliferation (**Fig. 3c**). Using quantitative reverse transcriptase real-time PCR, CK2α and CK2α' mRNA levels were found significantly decreased in TnFn cultured PC3-LN4 cells 24 and 48 h after treatment with s50-TBG-RNAi-CK2 (**Fig. 3d**), and the decrease was equivalent to that observed following treatment with TBG-siCK2 [31].

### Biodistribution of TBG nanocapsules

**Figure 4:**
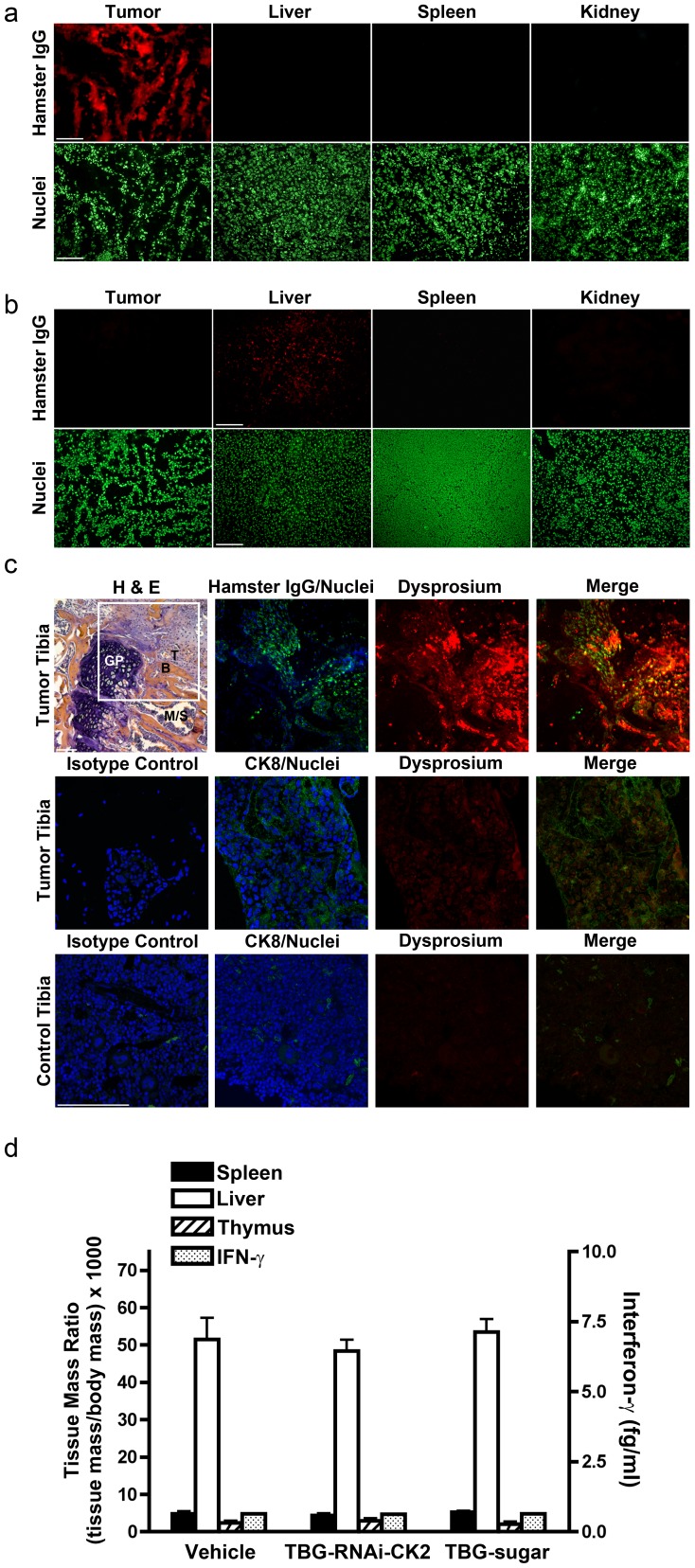
Physiological data for TBG nanocapsules – tissue uptake and analysis of early inflammatory response. (**a**) Binding of s50-TBG-RNAi-CK2 to tumor but not liver, spleen and kidney. Tissue sections were subjected to immunofluorescence analysis for Syrian hamster IgG following incubation with s50-TBG-RNAi-CK2. Scale bar 100 µm. (**b**) Binding of ASOR-DyDOTA to liver but not tumor, spleen and kidney. Tissues were subjected to immunofluorescence analysis for Syrian hamster IgG following incubation with ASOR-DyDOTA. Scale bar 100 µm. (**c**) Analysis of tibia bone for presence of tumor and uptake of TBG-DyDOTA nanocapsule 24 h following i.p. injection. Upper panels, tumor containing tibia: H&E stain, B = bone, GP = growth plate, M-S = marrow-sinus, T = tumor; immunofluorescence detection of Syrian hamster IgG (green); direct detection of Dy (red); merge of green and red. Center panels, tumor containing tibia: immunofluorescence detection of isotype control for CK8 (green); immunofluorescence detection of CK8 (green); direct detection of Dy (red); merge of green and red. Lower panels, mock injected tibia: immunofluorescence detection of isotype control for CK8 (green); immunofluorescence detection of CK8 (green); direct detection of Dy (red); merge of green and red. DNA counterstain is shown in blue. Scale bars 100 µm. (**d**) Analysis of liver, spleen and blood for inflammatory response. Immune-competent mice were injected i.v. with 10 mg/kg of s50-TBG-RNAi-CK2 or s50-TBG-sugar or with equal volume vehicle and tissues were collected after 24 h. Liver, spleen and thymus mass are presented relative to the mouse body weight (left axis). Interferon-γ was measured in blood serum (right axis).

To demonstrate that s50-TBG nanocapsules bind malignant and not normal tissue cells, and that they do not accumulate non-specifically in the reticuloendothelial system, frozen sections of PC3-LN4 orthotopic xenograft tumor as well as non-tumor bearing mouse liver, kidney and spleen were subjected to nanocapsule binding assays. In these assays, tissue sections were incubated with s50-TBG-RNAi-CK2 nanocapsules containing Syrian hamster IgG included in the protein shell. Following wash steps, the sections were processed for indirect immunodetection of the hamster IgG. Data in **Figure 4a** and Figure S1 demonstrate the binding of s50-TBG-RNAi-CK2 nanocapsules to tumor tissue, but not to liver, kidney or spleen. Further proof of ligand-induced nanocapsule tissue specificity is shown in **Figure 4b** and Figure S2 in which asialoorosomucoid (ASOR) nanocapsules with Syrian hamster IgG included in the shell were used to perform nanocapsule binding assays. ASOR is the ligand for the asialoglycoportein receptor expressed in hepatocytes, and the data demonstrate that the ASOR nanocapsule specifically binds to liver and not tumor tissue.

To provide validation for s50-TBG nanocapsule homing to malignant cells within mice, we initiated prostate cancer xenografts in bone by direct injection of PC3-LN4 cells into the tibia. The contralateral tibia in each mouse was mock injected. Once tumor growth was evident, the mice were injected with s50-TBG nanocapsules containing dysprosium-DOTA-dextran as the cargo (s50-TBG-DyDOTA) and collected after 24 h for processing. Tumor presence in bone was verified by H & E stain. The localization of s50-TBG-DyDOTA nanocapsules to tumor cells was verified by direct visualization of Dy, a fluorescent lanthanide element, and indirect detection of Syrian hamster IgG. Antibodies to CK8 were used to detect epithelial tumor cells. The data in **Figure 4c** depict the presence of Dy signal, representing the nanocapsule cargo, in tumor cells which specifically co-localizes with either Syrian hamster IgG included in the nanocapsule shell (**Fig. 4c** **, upper row**) or with CK8 representing the human prostate cancer cells (**Fig. 4c** **, center row**). Minimal Dy and CK8 signals were detected in mock-injected non-tumor bone from the same mice (**Fig. 4c** **, lower row**) which may be due to migration of cells from the xenograft tumor in the contralateral tibiae.

### s50-TBG nanocapsule-delivered nucleic acids do not induce early inflammatory response

Because an interferon or early inflammatory response is a serious issue for administration of particle suspensions containing nucleic acids, we measured the concentration of interferon-γ in serum and the tissue weight ratios for spleen, liver, and thymus in a cohort of non-tumor-bearing, immunocompetent outbred mice. These tissues are considered primary draining sites for intravenous formulations [35]. The results indicated no notable differences in the spleen, liver, or thymus weights for the animals treated with the s50-TBG nanocapsules containing RNAi-CK2 or trehalose relative to vehicle control (**Fig. 4d**). We also found no evidence of interferon-γ elevation, which typically is observed in particle-mediated early inflammatory responses (**Fig. 4d**) [36].

### Proof-of-concept for therapeutic efficacy and RNAi mechanism of s50-TBG-RNAi-CK2 *in vivo*

**Figure 5:**
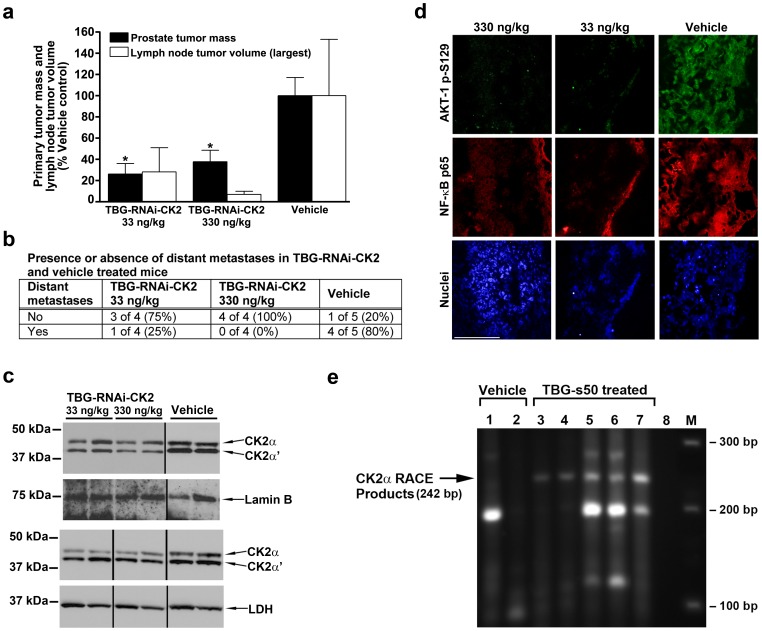
Proof-of-concept for therapeutic efficacy and mechanism of s50-TBG-RNAi-CK2 in orthotopic and metastatic prostate cancer xenograft tumors. (**a**) Primary tumor mass and lymph node tumor volumes are shown following s50-TBG-RNAi-CK2 treatments at 33 and 330 ng/kg compared to vehicle. Means ± SE are presented (TBG-RNAi-CK2 n = 4; Vehicle n = 5). * p<0.05. (**b**) Proportion of mice with distant metastatic tumors following s50-TBG-RNAi-CK2 treatments at 33 and 330 ng/kg compared to vehicle. Comparison using Fisher's Exact Test p = 0.046. (**c**) Immunoblot analysis for CK2α and CK2α' response in primary tumors. The upper panels represent 10 µg of nuclear matrix lysate with normalization to lamin B1. The lower panels represent 20 µg of cytosolic lysate with normalization to lactate dehydrogenase (LDH). (**d**) Protein and signaling response expression analysis in representative lymph node tumors. Lymph node tumor frozen sections were analyzed by indirect immunofluorescence co-staining for AKT-1 phospho-S129 (green) and NF-κB p65 (red). DNA counterstain is shown (blue). Scale bar 100 µm (**e**) CK2α RISC cleavage products are present in s50-TBG-RNAi-CK2 treated tumors. Total RNA was isolated from tumor tissue and used for 5′-ligation mediated RACE to determine if RISC mediated cleavage of the CK2α transcript occurred. Lanes 1 & 2, day-10 vehicle-treated flank tumors; Lane 3, day-5 s50-TBG-RNAi-CK2 treated flank tumor; Lane 4, day-6 s50-TBG-RNAi-CK2 treated flank tumor; Lane 5, day-10 s50-TBG-RNAi-CK2 treated flank tumor; Lane 6, day-10 s50-TBG-siCK2 treated flank tumor; Lane 7, day-6 s50-TBG-RNAi-CK2 treated orthotopic tumor; Lane 8, no cDNA water control; M, DNA size markers. The predicted 242 bp RACE product is indicated at left, the size in bp of the DNA standards shown on the right and the treatment administered indicated above the lanes.

Pilot acute dose-response experiments were carried out using s50-TBG-RNAi-CK2 to treat PC3-LN4 orthotopic prostate tumors in nude mice. After 10 to 14 days, orthotopically initiated tumors were palpable, and the mice were divided into treatment groups. Mice received two i.p. injections of either s50-TBG-RNAi-CK2 (4 mice per group) or vehicle (5 mice), given at an interval of 24 h. Thirteen days following the first treatment, the tumor tissue was harvested for analysis. Both of the dose levels 33 and 330 ng/kg resulted in greater than 50% lower tumor mass compared to vehicle (p = 0.011 and p = 0.029, respectively; **Fig. 5a**). Further, the treatments resulted in reduced volume of the largest collected retro-peritoneal lymph node tumor per mouse compared to vehicle as well as decreased mean length of all collected retroperitoneal lymph node tumors for dose levels 33 and 330 ng/kg (2.0±0.7 and 1.5±0.3, respectively) compared to vehicle control (3.6±0.7). Finally, there was a significant difference in the presence or absence of distant metastases when comparing s50-TBG-RNAi-CK2 to vehicle treated mice (p = 0.046; **Fig. 5b**). The orthotopic location of the primary tumors precluded precise measurement of initial tumor volumes, thus the inability to accurately determine starting tumor sizes may explain why the observed primary tumor response to 33 ng/kg was greater than that to 330 ng/kg.

To evaluate the CK2 target response, quantitative real-time reverse-transcriptase PCR analysis was performed on tumor RNA. The data demonstrated CK2α mRNA steady-state levels were reduced by 11 to 14% and CK2α' by 3 to 6% relative to vehicle controls. The limited reduction in CK2 mRNA levels is likely due to the time of sample collection, i.e., 11 days after the second treatment. Tumor tissue was lysed and fractionated into nuclear matrix and cytosol for immunoblot analyses. Representative results for tumors from each group are shown in **Figure 5c** **.** The density of the bands for CK2α and CK2α' proteins were calculated for all tumors and in the nuclear matrix fraction average CK2α protein levels of 1.06±0.19 for 33 ng/kg and 0.68±0.06 for 330 ng/kg, and average CK2α' protein levels of 0.83±0.17 and 0.43±0.03 for 33 and 330 ng/kg, respectively, were observed relative to controls (**Fig. 5c** **, upper panels**). At the highest dose level, both CK2α and CK2α' protein expression levels were similarly decreased (CK2α 0.51±0.12; CK2α' 0.39±0.11) in the cytosolic compartment as well (**Fig. 5c** **, lower panels**).

Lymph node tumors were analyzed by indirect immunodetection for CK2-related signaling response in the AKT and NF-κB pathways. Greatly reduced phosphorylation of the CK2-specific AKT-1 S129[37] site was observed after treatment with either 33 ng/kg or 330 ng/kg s50-TBG-RNA-CK2 (**Fig. 5d**, upper panels). Similarly, a dramatic loss of NF-κB p65 total protein was also detected, especially at 330 ng/kg TBG-RNAi-CK2 (**Fig. 5d**, center panels).

RNA from s50-TBG-RNAi-CK2 treated tumors on days 5, 6 and 10 from an independent experiment was analyzed using the modified 5′ RACE technique to map potential cleavage sites. RNA from a s50-TBG-siCK2 treated tumor as well as from control tumors were included as comparators. Day 5 and 6 tumors represent 2 treatments of s50-TBG-RNAi-CK2 given on days 1 and 4. Day 10 tumors represent 3 treatments of either s50-TBG-RNAi-CK2 or -siCK2 on days 1, 4 and 7. The data indicate that CK2α RISC cleavage products are detected on days 6 and 10 (**Fig. 5e**). In contrast to cultured cells, the CK2α' cleavage product was not detected. RACE products were sequenced for both s50-TBG-RNAi-CK2 and -siCK2 treated tumors, and the cleavage site detected was the same as shown in **Fig. 2c**. Reduced CK2α mRNA expression of 10 to 20% in the day-6 and day-10 tumors was verified.

## Discussion

We previously suggested the promise and adaptability of this tumor-directed nucleic acid based therapy targeting CK2 in both prostate cancer and in head and neck squamous cell carcinoma [14], [15], [30], [38], [39]. Here we have provided data on the protection of an oligomer cargo by TBG nanoencapsulation, malignant cell binding or uptake of TBG nanocapsules into cultured cells and into relevant prostate cancer sites in mice, possible and observed mechanisms of action for a single stranded DNA/RNA chimeric RNAi oligomer, and proof-of-concept pilot mouse xenograft therapy data.

In the pilot therapy study, we observed efficacy using s50-TBG-RNAi-CK2 in an *in vivo* PCa model at relatively low dosing of 330 ng/kg, representing just two treatments. At this dose level, effects on primary tumor weight and metastatic tumor formation and volume were apparent and concordant with decreased CK2 expression and signaling. Our 2-dose results compare favorably to another report of low-dose *in vivo* gene silencing in which non-targeted lipid-based siRNA delivery decreased target protein expression after a single dose of 3 µg/kg [40]. In comparison to other tumor-targeted siRNA delivery systems, our acute treatment study achieved reduced target protein expression and tumor weight at much lower dose levels [5], [6]. We used a vehicle control in this particular study to provide proof-of-concept that TBG-RNAi-CK2 could produce an appropriate therapeutic response in xenograft prostate cancer tumors. Subsequently, we conducted a separate study designed to identify an oligomer encapsulated in TBG which could serve a control for the encapsulated therapeutic RNAi. This study was conducted using flank tumors to facilitate direct measurement of tumor volume. Comparison of RNAi oligomers relative to vehicle control led to identification of a suitable control in PCa, and the comparative results demonstrated that both the RNAi and vehicle controls gave analogous results (i.e., tumor volume changes fully overlap). Thus, the results shown in Fig. 5c provide the proof-of-concept that TBG-RNAi-CK2 is capable of inducing tumor cell death *in vivo* which is attributable to the CK2 target. Further, the PC3-LN4 cells used in these studies are a model for PTEN-null, androgen receptor (AR) non-expressing, and androgen-independent metastatic PCa. While AR signaling is considered important for PCa cell survival, regardless of castration resistant phenotype [41], [42], the PC3-LN4 xenograft tumors used for these studies served to provide proof-of-concept data.

Use of single-stranded oligomers for RNAi activity in cultured cells and in mice has been previously documented [43], [44]. Moreover, it was shown that substitution of DNA residues into the 5′ end of the guide strand (i.e., seed region positions 2–8) produces an RNAi molecule with gene silencing activity and minimal off-target effects; whereas RNA residues in the 3′ end of the guide strand are essential [45]. Use of our DNA/RNA chimeric single-stranded RNAi-CK2 oligomer combines the efficiency of delivering just the guide strand with the potential reduction of off-target effects due to absence of a passenger strand. We demonstrated by *in vitro* experiments that both antisense- and siRNA silencing mechanisms were induced by RNAi-CK2; further, after use of s50-TBG-RNAi-CK2 to treat xenograft tumors, we showed that RISC cleaved CK2α mRNA was recovered 2 to 3 days post-treatment. We did not detect CK2α' cleavage product in treated tumors, although CK2α' protein levels decreased. This suggests that the RNAi-CK2 oligomers which contain 1 mismatch to human CK2α' mRNA and 2 mismatches to mouse stromal cell CK2α' mRNA may primarily be inducing RNase H- rather than RISC-mediated cleavage of this transcript *in vivo*. This notion is supported by the substantially reduced abundance of the CK2α' cleavage product observed with either siRNA-CK2 or RNAi-CK2 in the cell culture studies. Alternately, RNAi-CK2 may be blocking translation of CK2α or CK2α' transcripts into protein. Thus, our oligomer design allows for the possible coopting of multiple pathways to down-regulate target gene expression within the cell.

The present nanocapsule design, in which a reverse micelle composed of nucleic acid oligomers and protein is trapped into a salt crystal, results in a final product with average size of 15 to 20 nm. This is a favorable size for targeted systemic use as particles below 6 nm are rapidly cleared from circulation by kidney filtration and those greater than 50–100 nm exhibit significantly enhanced reticuloendothelial system accumulation [46]. The route of cellular uptake for the TBG nanocapsule as well as the trafficking of the delivered oligomers might also account for its efficacy [47]. In a paper focused on HNSCC, we presented data showing that the TBG nanocapsule enters cells using caveolae-mediated endocytosis [15]. The gene-targeting effectiveness of RNAi-CK2 oligomer delivery into tumor cells via tenascin receptors may be analogous to that observed for RGD peptide-conjugated antisense oligomers entering melanoma cells *via* the αvβ3 integrin receptors where introduction of RGD oligomers produced a greater effect than unconjugated oligomers [48]. Importantly, our nanoencapsulation technology is a water-based process which may help to preserve biologic functionality of the protein ligand shell. We hypothesize that the presentation of multiple TBG ligands on the surface of the nanocapsule promotes cancer cell specificity [46], which was demonstrated by the accumulation of s50-TBG nanocapsules in epithelial tumor cells within bone, with no observed accumulation or binding in non-transformed bone cells or other tissues such as liver, spleen or kidney. Quantitative evidence of specificity for tumors versus non-malignant tissues was presented recently [15].

While immunogenicity of a ligand-targeted particle cannot be fully evaluated outside of repeat dosing studies in primates, absence of an early immune response as demonstrated here has been found to be an important and very positive attribute of protein formulations with low immunogenicity [49]. Furthermore, we have previously demonstrated specific homing of s50-TBG nanocapsules to TRAMP orthotopic mouse prostate tumors, indicating that the human protein-composed TBG nanocapsules are recognized by tenascin receptors of mouse origin as well [16]. Finally, the TBG domains of tenascin-C in human and mouse exhibit 93% perfect identity and 99% positive identity, suggesting that we would detect adverse events arising in mice if induced by binding of the TBG nanocapsules to normal mouse tissues [16].

In conclusion, we have demonstrated the use of a single stranded DNA/RNA chimeric oligomer which is capable of using multiple pathways for gene silencing *in vivo*. Delivery of the RNAi-CK2 oligomer using the s50-TBG nanoencapsulation technology overcomes many of the challenges for nanomedicine [50] and demonstrates unique utility for malignant cell specific targeting *in vivo*. Further, downregulation of CK2 is equally effective regardless of prostate tumor cell androgen-sensitivity [16], [31]. This technology combined with the bi-functional chimeric oligomer directed against our therapeutic target CK2αα' holds great promise for treatment of PCa in both primary and metastatic locations.

## Materials and Methods

### Cell lines

PC3-LN4, C4-2 and BPH-1 cells were obtained and maintained as described [31]. All cells had undetectable levels of mycoplasma. The PC3-LN4 cell line was authenticated by the Genetic Resources Core Facility at Johns Hopkins University. The C4-2 and BPH-1 cell lines were not authenticated.

### Oligonucleotides

Oligomers were obtained from TriLink Biotechnologies (San Diego, CA). The RNAi-CK2-6R oligomer sequence is 5′-ATACAACCCAAACTccacau-propyl-3′; the RNAi-CK2-12R sequence is 5′-ATACAACCcaaacucacau-propyl-3′; the RNAi-RFP-6R (Red Fluorescence Protein) sequence is 5′-AGCTGCACGGGCTucuugg-propyl-3′. In these molecules the DNA nucleotides (uppercase) are standard phosphodiester linkages and the RNA nucleotides (lowercase) are 2′-*O*-methyl modified.

### Nanocapsule preparation and characterization

For s50-TBG nanocapsules containing oligomers or trehalose, a dispersion atomization method was used to package the cargo into nanocapsules composed of TBG as described [30]. Physical characterization of particles including average particle size measured by Transmission Electron Microscopy and surface charge determination employed published methods [51].

### DNase and proteinase K digestion of nanocapsules

Fig. 1c, left panel: 1.0 µg of RNAi-CK2 oligomer was digested for 24, 48, 72 or 96 h at 56°C with Proteinase K using RQ1 buffer system (Promega) adding fresh aliquots of enzyme every 12 h. The digests were then extracted with phenol:chloroform:isoamyl alcohol mixture and concentrated by ethanol precipitation. The precipitated material was resuspended in formamide loading buffer and subjected to electrophoresis on a TBE/20% polyacrylamide gel. The gel was stained using SybrGold. Fig 1c, right panel: Nanocapsules were incubated with Bio-beads (SM-2, BioRad) for 2 h rotating at 25°C to remove surfactant. Bio-beads were removed by centrifugation. 2.5 µg aliquots of naked RNAi-CK2 or s50-TBG-RNAi-CK2 were incubated overnight at 37°C in the absence or presence of 10 U of RQ1 DNase (Promega). Digestion was stopped with 2 mM EGTA (pH 8.0) and incubation at 65°C. Proteinase K digestion was performed for a total of 96 h at 56°C using RQ1 buffer system (Promega) with addition of fresh aliquots of enzyme every 12 h. Samples were concentrated using a speed-vac, separated by electrophoresis through a TBE/4-20% polyacrylamide gel, and stained using ethidium bromide.

### RNase H1 cleavage analysis

The RNA (5′-auguggaguuuggguuguau-3′) and phosphorothioate linked AS-CK2 (5′-ATACAACCCAAACTCCACAT-3′) oligomers were obtained from Integrated DNA Technologies. The chimeric phosphodiester RNAi-CK2-6R and RNAi-CK2-12R oligomers are described above. The RNA oligonucleotide was 5′ ^32^P-end-labeled using T4-polynucleotide kinase (NEB) and γ-^32^P-ATP (NEN). Labeled RNA was annealed with the RNAi-CK2-6R or RNAi-CK2-12R or AS-CK2 oligomers, then incubated for 0, 30, 60 or 120 seconds with E. coli RNase H1 (USB) as previously described [52]. The sizing ladders were generated by subjecting the 5′ end-labeled RNA oligonucleotide to alkaline lysis or T1 RNase (USB). The resulting digests and ladders were separated by running 20% 19∶1 urea PAGE and autoradiographs generated using Kodak intensifying screens and X-OMAT film.

### Cell proliferation assays

Proliferation assays were carried out as described [31], and each experiment was performed at least 3 times.

### TBG-FeO-dextran uptake

PC3-LN4 or BPH-1 cells (5×10^5^) were plated onto nanofiber scaffolds (Donaldson, Inc.) coated with 0.5 µg/cm^2^ of 2∶1 (w/w) tenascin-C (Millipore):Fibronectin-1 (Sigma-Aldrich) or laminin (Sigma-Aldrich), respectively. The next day, cells were treated with s50-TBG-FeO-dextran (1.25 µg of Fe) in 1 ml of media. Treatment of cultures was staggered so that exposure times varied from 2 to 24 h. Cultures were fixed in 2% paraformaldehyde, developed with DAB-enhanced Prussian blue for iron and counterstained with Fast Red. Images were acquired using an Olympus BX60 fluorescent microscope 40× objective with a digital color Q Imaging Retiga 2000R Fast1394 camera as 8 bit RGB 1600×1200 pixels. Experiment was performed 4 times.

### Animals

Male athymic CD-1 nude (Nu/Nu) mice (obtained from Charles River) or NOD-SCID-Gamma (NSG; VA internal breeding colony) were maintained under pathogen-free conditions. Nude mice were used at age 8–12 weeks. NSG mice were used at age 5 months. Mice were housed 4 mice per cage in autoclaved individually ventilated caging units with corn cob and crinkle cut paper bedding and ad libitum access to food (Harlan 2918 irradiated rodent chow) and autoclaved water. A 12 h light/dark cycle was used and average room temperature was 72°C. Experiments in this work were proof-of-concept pilot studies, and mouse numbers were not intended for therapeutic statistical significance.

### Ethics Statement

Facilities at the Minneapolis Veterans Affairs Health Care System were approved by the Association for the Assessment and Accreditation of Laboratory Animal Care International in accordance with the current regulations and standards of the United States Department of Agriculture (USDA) and National Institutes of Health (NIH) U.S. Department of Health and Human Services (DHHS). Animal experimental protocols were approved to be conducted at the Minneapolis Veterans Affairs Health Care System in strict accordance with the recommendations in the Guide for the Care and Use of Laboratory Animals of the National Institutes of Health. The protocols were approved by the Minneapolis Veterans Affairs Health Care System Institutional Animal Care and Use Committee (protocol 131101) and by the University of Minnesota Institutional Animal Care and Use Committee (protocol 1312-31154A). All surgery, as approved by the above-mentioned committee, was performed under inhaled isoflurane anesthesia, and all efforts were made to minimize suffering.

### In vivo therapy of orthotopic human prostate xenograft tumors in athymic nude mice

Orthotopic PC3-LN4 tumors were initiated as described [32]. Buprenorphine was administered by s.c. injection of 0.05 mg/kg twice per day for 2 days for analgesia per protocol. Mice were divided into treatment groups each containing small, medium and large tumors on the basis of manual assessment of tumor size. Groups of mice were subjected to intraperitoneal (i.p.) injection with 2 doses separated by 24 h of TBG-RNAi-CK2 in PBS/10% lactitol (4 mice per group) or vehicle alone (5 mice in group). Mice were sacrificed after 13 d of therapy, and both prostate and retro-peritoneal lymph node tumors were excised, measured, weighed following removal of necrotic material, and snap frozen in liquid nitrogen for RNA extraction and protein analysis or placed in OCT blocks. For the RACE analysis, mice were treated via tail vein with 10 µ/kg s50-TBG-RNAi-CK2 in PBS/10% lactitol on days 1, 4, and 7, and tumors were collected on either day 5, day 6, or day 10.

### Tibia bone tumors

Tumors were established in the bone of NSG male mice by injection of 0.5 to 1.0×10^6^ PC3-LN4 cells into the proximal end of the tibia bone using a 26-g needle and a twisting/drilling motion. The contralateral tibia of each mouse was mock-injected with a 26-g needle only. Buprenorphine was administered by s.c. injection of 0.05 mg/kg twice per day for 2 days for analgesia per protocol. Once tumors were palpable, the mice were injected i.p. with TBG-DyDota (100 nmol Dy/kg mouse). 24 h post-injection, both tumor and control tibiae were harvested and fixed overnight in 1× Z-fix (Anatech Ltd. #171) containing 7% sucrose. Tibiae were then placed in cassettes, rinsed 5× with tap water (1 L each time), and stirred at 4°C in 10-fold excess volume of 4 mM Tris-HCl/10% w/vol EDTA (pH 7.4) decalcification buffer. The decalcification buffer was exchanged twice per week until bones were soft (∼2 weeks). Tibiae were embedded into paraffin for sectioning. Four sets of tumor-containing and mock-injected tibiae were analyzed. Average mouse weight at start of experiment was 34.7 g; average weight at experiment end was 33.5 g.

### Immunoblot analysis of tumors

Approximately 0.1 mg of tumor tissue was homogenized in 1 ml of cytoskeleton (CSK) buffer. Nuclear matrix and cytosolic proteins were prepared and subjected to immunoblot analysis as previously described[32]. Antibodies used were: LDH-A (sc-27230) from Santa Cruz Biotechnology; CK2α (A300-197A) and CK2α' (A300-199A) from Bethyl Laboratories; Lamin B_1_ (33-2000) from Invitrogen.

### Purification of RNA and real-time RT-PCR

For cultured cells treated with nanocapsule, RNA purification, and quantitative real-time RT-PCR analysis were performed as described [31]. For tumor tissue, total RNA was isolated from approximately 40 mg of tissue as described [32]. The real-time reactions were run using TaqMan assays (Life Technologies: CSNK2A1 Hs00751002, CSNK2A2 Hs00176505, HPRT1 Hs01003267), 96 well FAST plates and an ABI 7900HT machine (Applied Biosystems Inc., Foster City, CA). Analyses were performed using ABI SDS 2.3 software. HPRT-1 was used as the reference gene for normalization. All results are reported as the average of reactions run using at least 2 different dilutions of cDNA.

### 5′ RNA ligase-mediated RACE

Total RNA was isolated from Deliver X Plus (Affymetrix) or Dharmafect D2 (Thermo Scientific) transfected PC3-LN4 cells or from PC3-LN4 xenograft tumor tissue using TRIzol (Life Technologies), and the quality of RNA was verified by gel electrophoresis. Total RNA (10 µg) from the tumors or 5 µg of total RNA from the cells were ligated with 0.5 µg of the RNA adaptor oligomer (5′-cgacuggagcacgaggacacugacauggacugaaggaguagaaa-3′) which contains the forward 5′ adaptor primer binding site in a 20 µL reaction using 20 U RNA ligase (New England Biolabs) and 40 U RNaseOUT (Life Technologies) according to the manufacturer's recommended conditions. The ligated RNA was purified by diafiltration (Millipore Ultracel-30K) using conditions for nucleic acids outlined by the manufacturer and the quality of the ligated RNA product was verified by gel electrophoresis. Six µL of the ligated RNA product were reverse transcribed using Superscript III Supermix (Life Technologies) and a CK2α gene specific primer (5′-CAAGGTGCTGATTTTCACTGTG-3′, IDT) or a CK2α′ gene specific primer (5′- GGTGTCTGTTCTCACTATGG-3′, IDT) designed to hybridize 3′ to the predicted RNAi-mediated cleavage sites in the respective transcripts. The resulting cDNAs (2 µL) were used for PCR using the forward (F) RNA adaptor primer (5′-GGACACTGACATGGACTGAAGGAGTA-3′) and reverse (R) CK2α (5′-TCACTGTGGACAAAGCGTTCCCATC-3′) or CK2α′ (5′- TGGATAAAGTTTTCCCAGCG-3′) gene specific primers. PCR was performed using Expand High Fidelity system (Roche Applied Science) using dNTP, buffer and enzyme concentrations recommended by the manufacturer. PCR was performed using 95°C for 3 min, 40 cycles of amplification (94°C for 45 sec, 57°C for 30 sec, 72°C for 45 sec), and 72°C for 10 min. PCR products were analyzed by 2% agarose Tris-borate-EDTA gel electrophoresis stained with ethidium bromide and visualized by UV light. Oligomers were obtained from Integrated DNA Technologies.

### Tissue-binding and immunofluorescence analyses

For all analyses, one day before immunofluorescence processing, antibodies were incubated at 4°C rocking overnight in a 50∶50 mixture of 0.025% tergitol (v/v) in PBS (PBS-NP):fish serum (SeaBlock, Thermo Scientific 37527), then spun through a Spin-X filter (Costar 8161) in a microcentrifuge for 5 min at 10,000 rpm.

Nanocapsule tissue-binding was tested on frozen tissue sections of liver, spleen, or kidney from 3 non-tumor mice or PC3-LN4 tumor from 3 tumor-bearing mice following incubation with s50-TBG-RNAi-CK2 for Fig. 4a or with ASOR-DyDOTA for Fig. 4b. Interaction of nanocapsule with tissue was detected by indirect capture of Syrian hamster IgG F(ab)_2_ fragment that was incorporated into the nanocapsule shell (s50-TBG-RNAi-CK2). For hamster IgG F(ab)_2_ fragment detection, either rabbit antibody to Syrian hamster (1∶25, Jackson ImmunoResearch 307-006-003) or mouse monoclonal antibody to Syrian hamster (1∶50, BD Pharmingen 554024) was used employing either streptavidin/biotin enhancement or the use of a horseradish peroxidase (HRP) bridging antibody. Briefly, the frozen sections were fixed and permeabilized with cold methanol for 10 min and washed twice in PBS. Sections were blocked for 30 min at room temperature in fish serum using the Vector Labs Avidin-Biotin kit in combination with 70 µg/ml of goat anti-mouse and anti-rat F(ab)_2_ fragments (Jackson ImmunoResearch), rinsed briefly in PBS-NP and incubated for 30 min with nanocapsules or buffer diluted in 1∶1 fish serum/PBS-NP. An omission control was included on every slide. Sections were then rinsed and incubated for 30 min at room temperature with antibody to Syrian hamster or buffer diluted in 1∶1 fish serum/PBS-NP. For this experiment there are 3 different omission controls included for every type of tissue; one incubated without the nanocapsule, one incubated without the primary antibody and one control incubated without either the nanocapsule or the primary antibody. Sections were then rinsed and sequentially incubated with either biotinylated donkey anti-mouse IgG F(ab)_2_ (1∶200, Jackson Immunoresearch) followed by streptavidin-Cy3 (1∶800, Jackson Immunoresearch) or goat anti-rabbit poly-HPO (1∶200, Thermo Scientific) and goat anti-HRP-Cy3 (1∶800 Jackson Immunoresearch) 30 min at room temperature for each step. Sections were counterstained with Sytox Green (1∶250,000, Life Technologies) for 10 min, washed and mounted in No Fade solution (50 mg p-phenylenediamine chloride in 5 ml PBS (Sigma)).

Indirect immunodetection for the NF-κB p65 and the AKT-1 phospho-S129 proteins in lymph node tumors was carried out on frozen sections using rabbit polyclonal antibody to AKT-1 phospho-S129 (1∶50, gift from L. Pinna) and a mouse monoclonal to NF-κB p65 (1∶50, sc-8008, Santa Cruz Biotechnology, Inc.). Briefly, slides were fixed and permeabilized with ice cold methanol for 10 min and washed twice in PBS. Sections were blocked for 30 min at room temperature in fish serum using the Vector Labs Avidin-Biotin kit in combination with 70 µg/ml of goat anti-mouse F(ab)_2_ fragments, rinsed briefly in PBS-NP, and incubated for 1 h at room temperature with combined primary antibodies diluted in 1∶1 fish serum PBS-NP. A primary antibody omission control was included on every slide. Sections were then rinsed in PBS-NP and incubated with biotinylated donkey anti-mouse IgG F(ab)_2_ (1∶200, Jackson Immunoresearch) or FITC-donkey anti-rabbit IgG F(ab)_2_ (1∶200 Jackson Immunoresearch) for 30 min at room temperature. Sections were rinsed in PBS-NP and incubated with either streptavidin-Cy3 (1∶800, Jackson Immunoresearch) or Alexa 488-goat anti-FITC IgG (1∶800, Life Technologies) at room temperature for 30 min. Sections were counterstained in 1 µg/ml bisbenzamide, washed and mounted in an anti-fade solution.

Indirect immunodetection of CK8 or Syrian hamster IgG and direct detection of Dy in bone was carried out on paraffin-embedded sections. Sections were deparaffinized and rehydrated through a xylene/ethanol series (3× Xylene, 3× absolute ethanol, 90% ethanol, 70% ethanol, 15 min each step). Slides were washed briefly with PBS, then incubated for 30 min in 20 mM citrate pH 7.0 at 37°C, washed briefly with PBS, then incubated in water for 2 days at 4°C. Sections were blocked for 30 min with 1% fish serum containing 5 mg/ml normal donkey serum, 1 mg/ml normal goat serum, and 4 drops of Avidin block per ml (Vector Labs) then washed briefly in PBS-NP. Syrian hamster F(ab)_2_ detection was performed as described above. For CK8 detection sections were incubated with 100 µL antibody mixture of mouse anti-CK8 (1∶30 Abcam ab27988) plus chicken anti-CK8 (1∶50 Genetex GTX 14053) overnight at 4°C with rotation. The IgG_1_ control antibody was used at 1∶50. Sections were washed 2× PBS-NP for 3 min and incubated with 100 µL antibody mixture anti-mouse poly-HPO (1∶100 Pierce) plus biotinylated anti-chicken (1∶200 Jackson Immunoresearch) for 30 min at room temperature. Sections were washed 2× PBS-NP for 3 min, and incubated with 100 µL antibody mixture goat anti-HPO-Cy3 (1∶800 Jackson Immunoresearch) plus streptavidin-Cy3 (1∶800 Jackson Immunoresearch) for 30 min at room temperature with slow rotation. Sections were washed 2× PBS-NP for 3 min and stained with Sytox Green diluted 1∶25,000 in TBS for 10 min at room temperature. Sections were washed 1× PBS-NP and 1× water for 5 min per wash and mounted as described above.

### Microscopy and image processing

Tissue-binding images were acquired using an Olympus BX60 fluorescent microscope 20× objective with a digital color Q Imaging Retiga 2000R Fast1394 camera as 8 bit RGB and 1600 x 1200 pixels. Bone images were acquired using a Nikon A1 Spectral Confocal microscope 60× water immersion objective with Nikon Elements software as 16 bit greyscale and 1024 x 1024 pixels. Lymph node images were captured using an Olympus BX61confocal microscope 40× objective with Fluoview 5.0 software as 8 bit RGB and 1024 x 1024 pixels. TIFF files were converted to 16 bits/channel using Adobe Photoshop. To process images, files were opened with ImageJ, channels or stacks were split and files saved as grayscale 16 bit files. Images and signals were adjusted as one group file with respect to their ‘no antibody control’ (background) reference. Adobe Photoshop adjustments: No gamma changes were made to any image. For Fig. 1b, the image was adjusted using curves changing input 90/output 100 and input 20/output 0. For Fig. 3a,b, color images were converted to greyscale and levels adjusted by input 250/output 200 and input 0/output 30. For Figs. 4a,b and 5d, pictures were colorized by assigning a color at 100% saturation. In Fig 4c - Sytox green grey scale images were processed using curves output 255/input 76, then converted to RGB and kept at 16 bit for color adjustment; CK8 grey scale images were processed using curves output 255/input 127, then converted to RGB and kept at 16 bit for color adjustment; Dysprosium grey scale images were processed using curves output 255/input 51, then converted to RGB and kept at 16 bit for color adjustment. Hue saturation was adjusted for Fig. 5d for red and blue to saturation +100 to try and balance the color saturation as viewed on multiple author's computer monitors.

### Tissue and IFNγ analysis

Immune-competent mice (3 per group) were injected i.v. with 10 mg/kg of s50-TBG-RNAi-CK2 or TBG-sugar or with equal volume vehicle and tissues were collected and weighed after 24 h. IFNγ levels in blood serum were analyzed at the University of Minnesota.

### Statistical analysis

Cell proliferation and qPCR expression level data were summarized and compared by treatment group using analysis of variance (ANOVA). Means ± SE are presented unless otherwise noted. The number of distant metastases was compared by treatment group using a Fisher's Exact test. P-values for pairwise comparisons were conservatively adjusted for multiple comparisons using a Bonferroni correction. P-values less than 0.05 were considered statistically significant.

## Supporting Information

Figure S1 **Binding of s50-TBG-RNAi-CK2 to tumor but not liver, spleen and kidney.** Tissue sections were subjected to indirect immunofluorescence analysis for Syrian hamster IgG following incubation with s50-TBG-RNAi-CK2. The tissues analyzed, Syrian hamster IgG detected, and DNA counterstain are indicated on the left. Scale bar 100 µm.
(TIF)

Figure S2 **Binding of s50-ASOR-DyDOTA to liver but not tumor, spleen and kidney.** Tissue sections were subjected to indirect immunofluorescence analysis for Syrian hamster IgG following incubation with s50-ASOR-DyDOTA. The tissues analyzed, Syrian hamster IgG detected, and DNA counterstain are indicated on the left. Scale bar 100 µm.
(TIF)

Checklist S1 **ARRIVE Guidelines checklist.**
(PDF)
