# Supplementary figures and images for: Tenfibgen Ligand Nanoencapsulation Delivers Bi-Functional Anti-CK2 RNAi Oligomer to Key Sites for Prostate Cancer Targeting Using Human Xenograft Tumors in Mice
Source: PLoS One. 2014 Oct 15;9(10):e109970. doi: 10.1371/journal.pone.0109970 (PMC4198192; doi:10.1371/journal.pone.0109970)

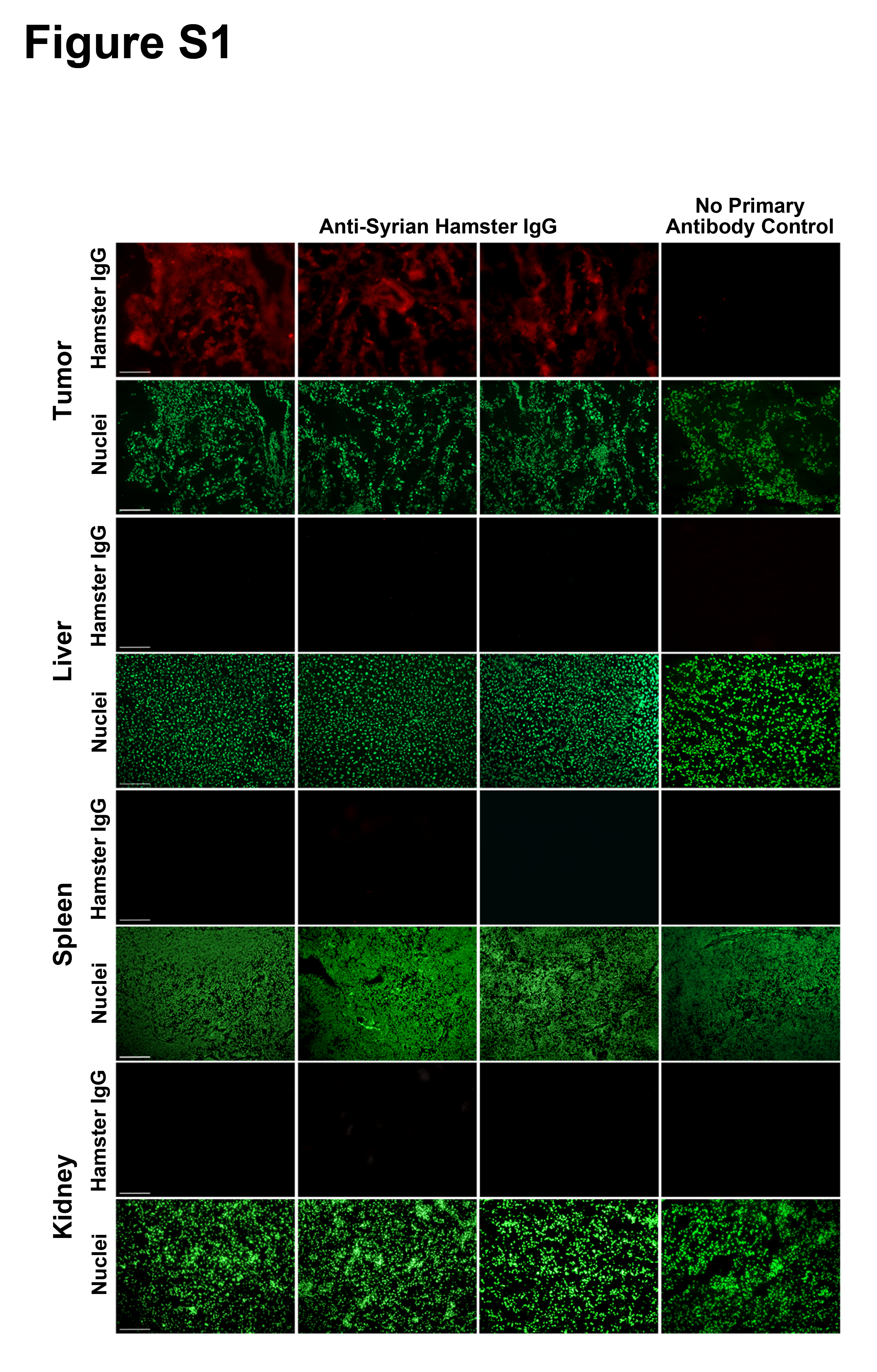

Supplement: Figure S1 — Binding of s50-TBG-RNAi-CK2 to tumor but not liver, spleen and kidney. Tissue sections were subjected to indirect immunofluorescence analysis for Syrian hamster IgG following incubation with s50-TBG-RNAi-CK2. The tissues analyzed, Syrian hamster IgG detected, and DNA counterstain are indicated on the left. Scale bar 100 µm. (TIF) [file pone.0109970.s001.tif]

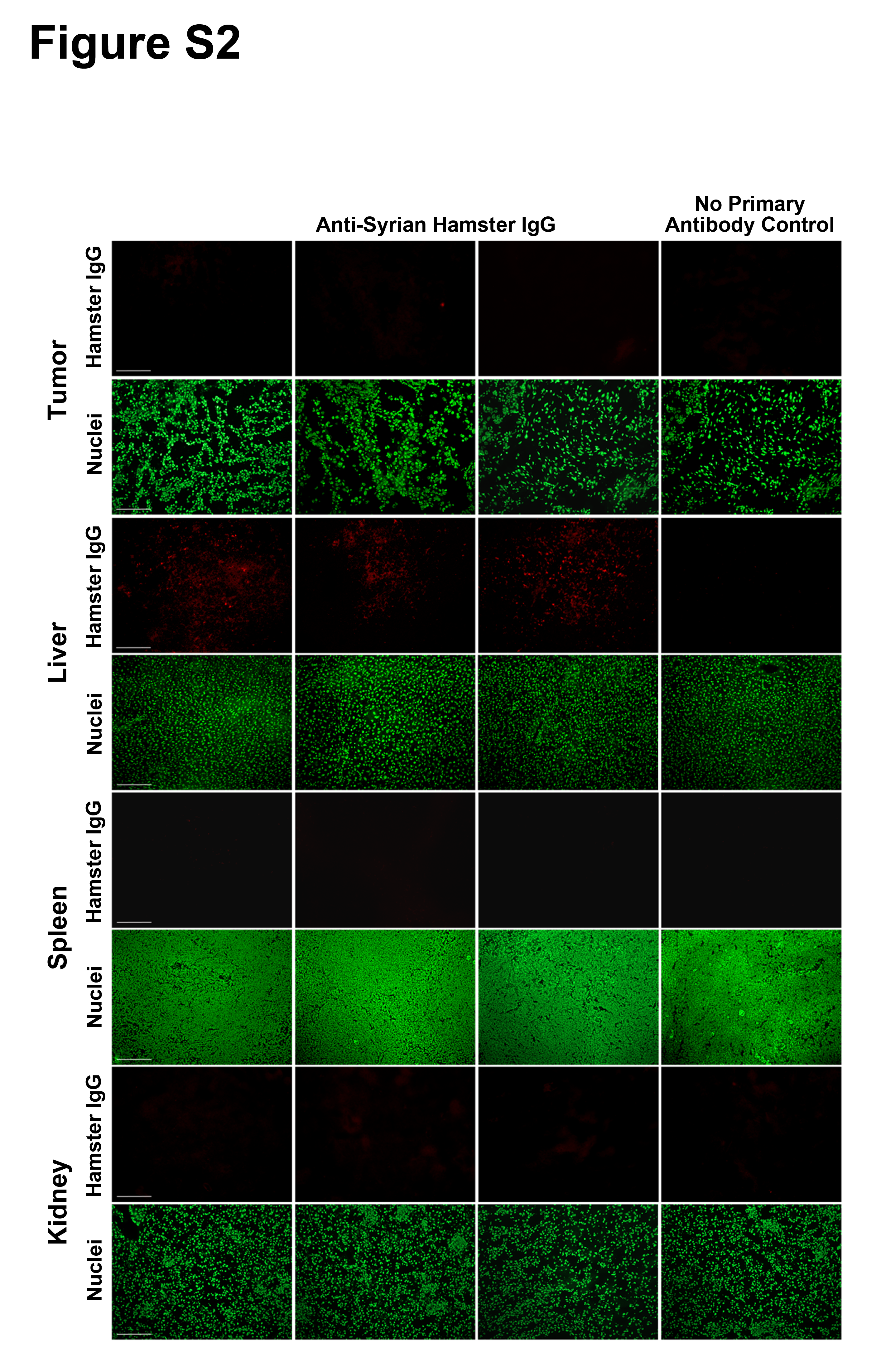

Supplement: Figure S2 — Binding of s50-ASOR-DyDOTA to liver but not tumor, spleen and kidney. Tissue sections were subjected to indirect immunofluorescence analysis for Syrian hamster IgG following incubation with s50-ASOR-DyDOTA. The tissues analyzed, Syrian hamster IgG detected, and DNA counterstain are indicated on the left. Scale bar 100 µm. (TIF) [file pone.0109970.s002.tif]
